# Food Safety Knowledge and Attitudes: A Cross-Sectional Study among Saudi Consumers from Food Trucks Owned by Productive Families

**DOI:** 10.3390/ijerph19074322

**Published:** 2022-04-04

**Authors:** Latifah A. Alhashim, Najim Z. Alshahrani, Amal M. Alshahrani, Shamsun Nahar Khalil, Muneera A. Alrubayii, Sarah K. Alateeq, Ossama Mohammed Zakaria

**Affiliations:** 1 College of Medicine, King Faisal University, Hofuf 31982, Saudi Arabia; 217016758@student.kfu.edu.sa (M.A.A.); 217017612@student.kfu.edu.sa (S.K.A.); 2 Department of Family and Community Medicine, College of Medicine, University of Jeddah, Jeddah 21589, Saudi Arabia; 3 General Directorate of Communicable Diseases Control, Ministry of Health, Riyadh 11176, Saudi Arabia; amal.jrais@gmail.com; 4 Department of Family and Community Medicine, College of Medicine, King Khalid University, Abha 62529, Saudi Arabia; shamsun203@gmail.com; 5 Division of Surgical Paediatrics, Department of Surgery, College of Medicine, King Faisal University, Hofuf 31982, Saudi Arabia; Ossama.m.zakaria@gmail.com

**Keywords:** food safety, foodborne diseases, food trucks, family producers, Saudi Arabia

## Abstract

Food businesses, such as food trucks (FTs) and family producers have grown rapidly and become popular with people in Saudi Arabia. As foodborne diseases are still a public health concern in the country, consumers should be aware of the safety of foods sold by food trucks. Since there is a dearth of literature, this study aimed to assess the knowledge and attitudes of consumers of food from FTs owned by family producers regarding food safety and foodborne disease. A cross-sectional study was conducted among 921 consumers of food from FTs from two cities in the southern region of Saudi Arabia. Data were collected using an online questionnaire from September to November 2021. Finally, multiple linear regression and a logistic regression model were used to identify the predictors of knowledge and attitude regarding food safety and foodborne illness, respectively. Overall, respondents had moderate knowledge and higher positive attitudes regarding food safety and foodborne illness. Respondents’ knowledge about the potential harm and attitudes towards the safety of foods sold by FTs were significantly associated with marital status, education level, and monthly family income. The findings of this study highlight the need to improve the knowledge, awareness, and attitudes of Saudi consumers of food from FTs owned by family producers on food safety and foodborne illness through intervention programs, particularly targeting consumers with lower educational attainments and income status, to control foodborne diseases.

## 1. Introduction

Foodborne diseases (FDs) have become a life-threatening concern for people worldwide and pose barriers to a country’s health system and socioeconomic development [1,2]. The World Health Organization (WHO) estimates consumption of unsafe food causes approximately 600 million cases of FD and 420,000 deaths every year [3,4]. These data indicate that 7.69% of the world’s population experiences FDs every year; moreover, mortality from foodborne illnesses represents 7.5% of total mortality [3,5]. The Eastern Mediterranean Region (EMR) is the third-highest region with FDs, estimated at over 100 million cases annually [6]. In 2020, Saudi Arabia registered 3081 cases of FDs with 1258 of them being in the age group of 15 to 45 years [7]. Unfortunately, the majority of individuals with FDs do not seek care from health facilities due to the acute gastrointestinal illness being self-limiting [8]. Consequently, in Saudi Arabia, FDs may directly and/or indirectly impact individuals’ health, wellbeing, and productivity, as well as the country’s health care structure and costs.

“Eating out” (i.e., consumption of outdoor food) is one of the contributing factors to foodborne illness [9,10]. It is evident that FDs were attributed to the initial contamination of ready-to-eat foods without any contribution from the final food handlers [10,11]. Moreover, lack of food hygiene, poor food handling practices, cross-contamination and inadequate storage are some of the major causes of foodborne illnesses, which are related to ready-to-eat outdoor foods [11]. Despite this, eating ready-to-eat outdoor food is flourishing in many countries, including Saudi Arabia, because of various reasons such as lack of time, hectic lifestyle, desire for food diversity, and better taste, affordability, and accessibility [4,12]. To meet the demand for ready-to-eat foods and to create employment, food trucks (FTs) have emerged and are expanding rapidly in Saudi Arabia as in other countries [13] such as the USA [14], Brazil [15], China [16], and Vietnam [17]. FTs are movable commercial kitchens similar to brick-and-mortar restaurants that offer ready-to-eat foods and provide services quickly [11]. Many family producers in Saudi Arabia are involved in the food business, usually operating via food trucks or using social media, offering traditional foods, frozen foods, as well as snacks, including samosas and pastries. However, the safety and hygiene of food in these FTs have increasingly come into question [11,13].

Given the itinerant nature of the FTs vehicles, effective food safety regulation and inspection are often evaded. In 2016, 9 out of 96 licensed food trucks in Boston were shut down when “on the spot” inspections had revealed food safety hazards [14]. A recent study conducted in the USA reported that food trucks could be a potential source of clinically relevant *E. coli* and *Salmonella* carrying intestinal pathogenic virulence factors or antibiotic resistance genes [15]. Previous studies conducted in China [16], Turkey [2], Malaysia [18], and Brazil [19] reported that foodborne disease outbreaks occurred due to poor hygiene and sanitary practices and the presence of contaminated food samples among FTs. In Vietnam, Nguyen et al. [17], through their study about food hygiene and safety standards on food handlers, stated there was a need to enhance customers’ protection systems, inspect and supervise the food processing progress by local authorities, as well as create awareness for food customers about food hygiene and safety standards. The risk and consequences of consuming street food or food from FTs are associated with several sociodemographic factors such as consumers’ education, age, and sex [20] and other factors including consumers’ knowledge about food safety, foodborne illness, and potential hazards [21].

Consumers’ knowledge regarding food safety and the foodborne illness of street food may positively impact their attitudes, which may subsequently translate into better food safety practices [22]. Similarly, Saudi consumers of food from FTs should be equipped with appropriate knowledge and perceptions regarding food safety issues to understand how to protect themselves against FDs. Although the current food safety and hygiene concerns from the ministries of health globally resonate with consumers’ preferences, the consumers’ involvement in inappropriate practices seems to have not changed, hence, standing a risk of foodborne diseases. In Saudi Arabia, a handful of studies have been conducted on food safety issues which were limited to specific population groups such as food handlers, food service staff, students, and mothers [23,24,25,26,27]. However, a study on knowledge and attitudes regarding food safety and foodborne illness among consumers of food from FTs owned by productive families in Saudi Arabia remains unexplored. To address this gap, an empirical study that evaluates the consumers’ knowledge and attitudes regarding food safety and foodborne diseases is urgently needed. Therefore, this study aimed to assess the knowledge and attitude of consumers of food from FTs owned by family producers regarding food safety and foodborne disease in a sample of Saudi Arabians.

## 2. Materials and Methods

### 2.1. Study Design, Settings, and Sample

The current cross-sectional study was conducted among consumers of food from FTs from two conveniently selected cities in the Asser region of Saudi Arabia, namely Abha and Khamis Mushit. At first, four cities in the Asser region of Saudi Arabia, namely Abha, Bisha, Khamis Mushit and Bareq, were selected by lottery techniques. The name of each city in the Asser region of Saudi Arabia was written on a piece of paper, folded, and entered into a container with other pieces of folded paper with the city names. A research staff picked four names from the container for the initial selection of study areas. Finally, the research team chose two cities (Abha and Khamis Mushit) due to the convenience of data collection and communication and the predominance of food truck business in these areas. The study period was about two months, from 20 October to 25 December 2021.

In Saudi Arabia, there are many types of FTs, some of these owned by restaurants or food companies. However, in the current study, we have focused solely on FTs owned by family producers. Since no strict rules and regulations are required from the government or any health agencies to introduce such FTs business in Saudi Arabia, food safety can be a major concern. The study included participants upon assessing the following eligibility criteria: (i) individuals aged ≥18 years (both male and female) who buy and consume ready-to-eat food from FTs made by Saudi family producers and (ii) Saudi citizens by birth. Non-Saudi consumers were excluded from the study. A purposive sampling technique was used to recruit the study participants. A minimum sample size of 422 participants was calculated using a single population proportion test [28]. The estimation was based on the following assumptions: (i) 50% prevalence of expected food safety knowledge and attitudes of consumers from FTs (since there were no similar studies in Saudi Arabia), (ii) 95% confidence interval (CI), (iii) 5% margin of error, and (iv) 10% non-response rate. To obtain higher external validity and greater generalizability of the study [29], we targeted collecting more samples than our calculated sample size. Finally, we included 921 samples in this study.

### 2.2. Data Collection Procedure

Due to the restrictions induced for COVID-19, such as movement restrictions and social distancing measures, we designed an online-based survey. Data were collected through a structured questionnaire using an online-based platform “Google template”. We barcoded the survey link, printed it on paper, and distributed it to the selected cities (*n* = 2) by hanging either side-by-side with food trucks or walls of the vending area (in case of a small booth). Trained medical students (*n* = 6) were responsible for distributing barcode-protected survey tools, and while distributing, they informed the sellers about the purposes and implications of this survey and asked them to encourage the buyers to fill it out voluntarily. Thus, the consumer who agreed scanned the barcode of the survey link via their phone devices and obtained the survey questionnaire. Before starting to fill out answers to the survey questions, informed consent was obtained from each respondent by clicking the consent statement. The informed consent statement for the respondents was as “I do hereby, after reading the research objectives, participate in the survey sharing my information by answering questions consciously and willingly.” After they completed the survey, they submitted it to our data pooling platform by clicking the option “submit”. The following screening question was introduced at the top of the questionnaire to identify the consumers of food from food truck vendors: “Did you ever buy ready-to-eat food from Food Trucks made by Saudi family producers?” The participants’ responses were anonymous and coded through unique serial numbers to avoid all possible identifying information such as names and contacts from publicly available data. Initially, the English version of the questionnaire was retrieved from the previous studies [2,17,30,31] that assessed knowledge and attitudes towards food safety among consumers and street food vendors. Then, the questionnaire was re-constructed based on the Saudi Arabian context by discussing with academic experts of relevant fields to ensure validity. The questionnaire was translated from English to Arabic (local language) by a bilingual person to enable easy understanding of the questions and avoid any questionnaire bias. Before administration of the final version of the questionnaire, a pretest was performed among randomly selected small groups of consumers (*n* = 10) of street FTs from Ahad-Rufidah city in the southern region to ensure the reliability and applicability of the questionnaire. The results of the piloted study were not included in the final analysis.

### 2.3. Study Variables and Measures

The questionnaire was comprised of four sections including (i) study objectives and consent letter, (ii) sociodemographic information, (iii) assessment of knowledge about the potential harms of foods sold by FTs, and (iv) attitudes towards the safety of foods sold by FTs (see Supplementary Materials).

#### 2.3.1. Outcome Variables

Consumers’ level of “knowledge” about the potential harms (5 items) and “attitudes” towards the safety of foods sold by FTs (13 items) were the dependent variables of this study. A set of five questions with three possible answers (e.g., “correct”, “incorrect”, and “don’t know”) was used to assess the participants’ knowledge about the potential harms of foods sold by FTs. The “knowledge about potential harm section” included information on foodborne illnesses, foodborne pathogens, and sources of food contamination. We included the “don’t know” options in the items to reduce the randomness and to avoid the possibility of correct answers by guessing. One point was assigned for each correct answer and zero for the other two answers (“incorrect” and “don’t know”). Total summation of the discrete scores of the different items was calculated for an overall score.

Participants’ attitudes towards the safety of foods sold by FTs were assessed by a 4-point Likert scale (agree = 3, neutral = 2, disagree = 1 and don’t know = 0), which contained 13 items. This section included participants’ attitudes on food hygiene, personal hygiene, cross-contamination, worker health status, and cleaning. Considering attitude, the composite mean score was calculated for each participant, which ranged from 1 to 3; a higher mean score indicated a more positive attitude. Participants with a composite mean attitude score of less than 2 were considered negative attitudes. The composite mean score of 2 to 2.5 was considered as neutral status, while the composite mean score of 2.6 to 3 was considered to be a positive attitude.

#### 2.3.2. Explanatory Variables

The explanatory variables included the participants’ sociodemographic information (age, gender, marital status, employment status, education level, and income status).

### 2.4. Data Analysis

Data were extracted, revised, and coded through Statistical Package for the Social Sciences (SPSS) software (IBM SPSS version 22, Inc., Chicago, IL, USA). Descriptive statistics (e.g., response frequencies/percentage, means, and standard deviations) were used to summarize variables of interest such as demographics. A one-way analysis of variance (ANOVA) and independent sample t-tests were applied to assess sociodemographic differences in the scores of participants’ knowledge about the potential harm of foods sold by FTs. A Chi-square test was used to determine the sociodemographic differences in participants’ attitudes towards the safety of foods sold by FTs. Finally, multiple linear and logistic regression analyses were performed to identify the determinants of participants’ knowledge and attitude towards food safety sold by the FTs, respectively. All statistical analysis was conducted using two-tailed tests, and a *p*-value less than 0.05 was considered statistically significant.

## 3. Results

### 3.1. Socio-Demographic Profile of Study Participants

Out of 921 participants, more than half (57.9%) were female, and two-thirds (63.8%) were single. Participants’ ages ranged from 18 to 64 years with a mean age of 24.9 (±11.6) years old. More than two-thirds (68.4%) of the respondents had an educational attainment of university level or above. Half (50.3%) of the participants reported working in a non-healthcare field, and below one-third (31.9%) worked in a healthcare field. Almost half (49%) of the participants reported having a monthly income of between 5000 and 15,000 Saudi Riyal (SR), followed by 37.2% and 13.8% who monthly earned >15,000 SR and >5000 SR, respectively (Table 1).

### 3.2. Knowledge Regarding Potential Harms of Foods Sold by the FTs

Assessment of participants’ level of knowledge about the potential harms of foods sold by the family producer-owned FTs is summarized in Table 2. The majority of the participants answered correctly about the knowledge items; “abortion in pregnant women can be induced by food-borne disease” (50.4%), “hepatitis A virus is a food-borne pathogen” (61.9%) and “buying from family producer-owned FTs increases the risk of food poisoning” (64.3%). However, more than one-third of participants (39%) wrongly reported that reheating cooked foods can contribute to food contamination (Table 2).

The overall mean knowledge score about the potential harms was 2.6 ± 1.3 out of 5 points. The average knowledge score regarding the potential harms significantly differed by participants’ age (*p* = 0.012), marital status (*p* = 0.001), and educational level (*p* = 0.003). The average score was significantly higher among older-aged participants than the younger aged group (2.29 for younger aged vs 2.51 for older aged; *p* = 0.001). The average score was 3.05 for separated or widow participants compared to 2.29 for single people (*p* = 0.001). The mean score was 2.46 for participants with an education level below secondary compared to 3.09 for university graduates (*p* = 0.004) (Table 1).

As shown in Table 3, when participants’ age, gender, marital status, nationality, education level, worker status, and monthly income were assessed on predicting consumer knowledge of potential harm using multiple linear regression, the overall statistic was not significant. Only marital status (the married) and a high education level significantly contributed to this prediction combination (Table 3).

### 3.3. Consumers’ Attitude Level towards the Safety of Foods Sold by FTs

**Figure 1:**
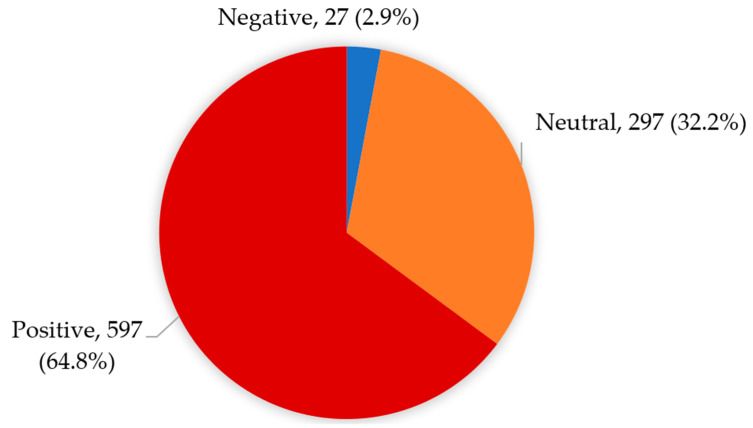
Overall attitude level of participants towards the safety of foods sold by the food trucks.

The overall attitude level of participants towards the safety of foods sold by the family producer-owned FTs is shown in Figure 1. Approximately, 64.8% of participants had a positive attitude towards food safety, while 32.2% had a neutral attitude (don’t care), but only 2.9% had a negative attitude (Figure 1).

As shown in Table 4, as far as participants’ attitude towards the safety of foods sold by FT is concerned, only monthly income was statistically significant on the Chi-square test. Participants who earned at least 15000 SR had a higher positive attitude (70%) compared to those who earned less (*p* = 021) (Table 4).

The association of participants’ attitudes towards the safety of foods sold by FTs across sociodemographic characteristics was further assessed using logistic regression. When each of the variables was independently assessed against participants’ attitudes towards the safety of foods sold by FTs, only monthly income was statistically significant (Table 5).

## 4. Discussion

The present study explored the knowledge and attitudes of consumers of food from family producer-owned FTs regarding food safety and foodborne disease in Saudi Arabia. Overall, moderate knowledge and positive attitudes regarding food safety and FDs were found among consumers of food from productive families-owned FTs in Saudi Arabia. This finding is consistent with previous studies conducted in Bangladesh [4] and Haiti [30] reporting that consumers of street foods had a moderate level of food safety knowledge. By these factors, the FTs consumers’ level of knowledge and attitudes regarding food safety and FDs would be expected to be reasonably high as asserted by Ma et al. [31]. Indeed, this study shows that the majority of the consumers of food from FTs had the required knowledge regarding food safety, which might help them to maintain healthy food choice and food hygiene practices and subsequently reduces the chances of getting FDs. Only 20% of these participants indicated not knowing how to reduce food-related infections. This high number of knowledgeable participants was partly enhanced by the high education level of the FT consumers observed above. This observation agrees with Ma et al.’s [31] in their study of food safety knowledge, attitudes, and behavior of street food vendors and consumers in China.

Aligning with findings from a previous study among food truck customers in the city of Curitiba, Brazil [32], the present study reported that more than half of the consumers of food from FTs were female. Unfortunately, this observation in line with the street food segment is considered not typical of the female sex. The FTs promote eating as a cultural activity of leisure and fun that would be more attractive to males than females [32]. Studies that have reported more males than females as FTs consumers have indeed supported this justification [2,33], although on the contrary, globalization and women emancipation could be a better explanation for this study’s sex finding. In their study, Liu and Niyongira [34] attributed it to women being the major meal planners who would take the food safety issue more seriously than the men.

Our study found that the majority of the FT consumers were young people (mean age 24.9 years), which is supported by previous studies [3,35]. Young people consume more of the out-of-home foods on the street than older age groups. Although Shin et al. [35] and Yoon and Chung [3] asserted that consuming food from FTs was primarily for fun, excitement, emotional values, and a pleasant social experience; in this study, the primary reasons in their ascending order of score for buying food from FTs were that the food was liked, promoted by social media, had better taste, and was cheap. For these reasons, the FTs were established to promote easy, fast, quick, and cheaper food that can be accessed along the city streets [2].

Our study reported that participants obtained a moderate level of knowledge score for the potential harm of food sold by FTs. This finding is comparable to previous studies conducted among consumers of street food [4,36]. In the present study, more than one-third of the respondents (39%) answered wrongly about reheating cooked food, which indicates consumers’ poor knowledge about food handling practices, food processing, and preservation. Appropriate handling practices of food and leftovers is an important aspect of consumer food safety [37,38]. Thus, the arrangement of a food safety education program among consumers as well as food vendors, incorporating the basics of food handling practices such as reheating, leftover food management, preservation techniques, and foodborne illness is highly recommended to improve food safety knowledge.

The findings of the current study showed that the average score about knowledge for the potential harm of food sold by FTs was significantly associated with participants’ marital status (being married) and education level (high education level). With regard to marital status, previous studies [39,40] have also found a statistically significant association between marital status and food safety knowledge, with those not married having a high knowledge score. This is the opposite of the present study’s findings, as those who were married had higher food safety knowledge scores. To obtain more insights and understand the reasons behind these discrepancies, a qualitative study is highly recommended.

Higher educated consumers had higher food safety knowledge scores—another key finding of our research. If a consumer attained at least a university education, knowing that FTs might carry potential harm was higher than those who attained less than secondary education. Our assertion agrees with that of Liu and Niyongira [34] and Hammoudi et al., [41] who stated that educated consumers pay more attention to food safety as compared to consumers with low education levels. Moreover, food consumers who buy safe food have more knowledge and are more willing to pay a higher price [38].

Additionally, environmental and hygienic factors of food trucks negatively influenced consumers’ attitudes and visit intentions toward the food truck dining experience. At the same time, hedonic benefit led to positive attitudes and visit intention [33]. The current study found that most of the participants (64.8%) had a positive attitude towards the food safety of the FTs, and positive attitudes were significantly influenced by a higher monthly income. This is possibly because their financial solvency might mean they could afford to buy food from FTs frequently; therefore, they were more willing to pay attention to food safety considerations at the FTs. It is important to note that street food such as that of FTs meets the consumption needs of all social and economic classes because it is relatively cheaper [11,42]. A recent study showed that affordability followed by personal preferences such as convenience or accessibility were the main reasons for consuming street-food among Bangladeshi consumers [4]. All these factors act as a driving force for consuming or attitudes towards the safety of foods sold by FTs. To understand how consumers’ knowledge of and attitudes toward food safety and foodborne illness may translate into practices, a followup investigation based on knowledge, attitudes, and practices (KAP) modeling is recommended.

Food trucks are considered a social innovation of the practice of eating out [43]. Collectively, in this study, we found consumers of food from family producer-owned FTs had considerable knowledge and higher positive attitudes regarding food safety and FDs. The exponential growth in the popularity of food trucks makes this food business sector more vulnerable to food safety issues. FTs are associated with potential risks that could result in foodborne illnesses [44]. Therefore, owners or sellers have an important role to play in maintaining the safety and quality of food sold by FTs. A previous study from the USA revealed a lack of food safety knowledge among food trucks owners/managers, with areas of improvement concerning personal hygiene, food preparation, cleaning and sanitizing, and safe chemical handling [45]. A recent study conducted in Jeddah city, Saudi Arabia reported that food safety knowledge was good among food vendors of street food trucks, and they also demonstrated a satisfactory level of hygienic practices [46]. Preliminary information on the food safety aspects of family producer-owned FTs, such as evaluating owners/managers’ status of food safety knowledge, attitudes, and practices as well as related factors, is urgently needed which will serve as a starting point for future food safety interventions.

Our findings recommend improving the knowledge, awareness, and attitudes of Saudi consumers of food from family producer-owned FTs on food safety and foodborne illness through related education and interventions (such as food safety training, awareness campaigns, publicity through social media, etc.), particularly targeting young adult females, individuals with lower educational and income status, to control foodborne diseases and any hazards related to the consumption of food from FTs. The food truck industry is rapidly growing in Saudi Arabia, but the policies governing this sector of the food service business are inconsistent or lack enforcement. A recent qualitative study in the USA reported that food safety training is necessary and essential for a food truck business [47]. Food truck owners’ perceived time, money, and lack of appropriate facilities are considered potential barriers for providing training to their employees [47]. Thus, the Saudi Food and Drug Authority (SFDA) should make concerted efforts to maintain the safety of foods sold by family producer-owned FTs and improve the knowledge and level of practices among food vendors of FTs through targeted educational campaigns, food safety training, and strict monitoring and regulation. Such policy would obligate food vendors to maintain food safety and hygiene standards, thereby reducing foodborne illness risk. Therefore, further studies on food safety and hygienic issues among owners of FTs are highly recommended to understand whether they are aware of food safety and foodborne illness.

The study has several strengths. It was one of the first studies to explore food safety and foodborne illness-related knowledge and attitudes among consumers of food from FTs owned by family producers in Saudi Arabia. Being the first investigation, the findings may provide baseline data for policymakers and public health practitioners to design and implement an evidence-based intervention across the country to improve consumers’ knowledge and attitudes on food safety and foodborne illness. Moreover, a larger sample size represents an additional potency in this study. However, there are some shortcomings in this study that need to be acknowledged when interpreting the findings. Due to the cross-sectional design, this study does not allow us to establish a causal relationship. Further studies that incorporate a longitudinal design would provide causal interference for consumers’ knowledge and attitudes towards food safety issues. The study was conducted in only two cities of the Asser region of Saudi Arabia (Abha and Khamis Mushit), so the findings cannot be generalized across the country. We gathered data through online survey links, which might create a response barrier for some respondents. Furthermore, respondents’ self-reported responses may occur information and reporting bias.

## 5. Conclusions

This study revealed that Saudi consumers of food from family producer-owned FTs had moderate knowledge and higher positive attitudes regarding food safety and FDs. Several sociodemographic factors were associated with participants’ knowledge of potential harm (marital status and education level) and attitudes towards the safety of foods sold by FTs (income). Given the associated public health risk that can result from inadequate or a lack of food safety, there is a need for targeted strategies and intervention programs, such as educating about foodborne pathogens and their adverse impact, food safety training, and awareness campaigns to improve consumers’ as well as food vendors’ knowledge and attitudes regarding food safety and FDs. It is imperative to conduct more extensive research on both consumers and food sellers to gather a holistic picture of their level of knowledge, attitudes, and practices regarding food safety and FDs. Moreover, further studies which may incorporate a longitudinal approach or microbial analysis of food samples are warranted to understand links between consumption of foods from FTs and disease occurrence.

## Figures and Tables

**Table 1:** Background characteristics and distribution of participants’ knowledge regarding potential harms of foods sold by the family producer-owned food trucks (*n* = 921).

Factors		Harm Knowledge Score		*p*-Value
	Frequency (%)	Mean	SD	
Age in years 18–25				0.012 *
	482 (52.3%)	2.35	1.20	
26–34	217 (23.6%)	2.48	1.38	
35–44	134 (14.5%)	2.78	1.52	
45–54	70 (7.6%)	2.60	1.42	
55+	18 (2.0%)	2.17	1.50	
Gender				0.254 ^$^
Male	388 (42.1%)	2.52	1.38	
Female	533 (57.9%)	2.41	1.29	
Marital status				0.001 *
Single	588 (63.8%)	2.33	1.22	
Married	281 (30.5%)	2.60	1.43	
Divorced/widow	52 (5.6%)	3.08	1.64	
Educational level				0.003 *
Below secondary	44 (4.8%)	2.46	1.64	
Secondary	247 (26.8%)	2.34	1.26	
University/above	630 (68.4%)	3.09	1.32	
Job title				0.653
Not employed	126 (13.7%)	2.48	1.37	
Non-healthcare workers	463 (50.2%)	2.45	1.35	
Healthcare workers	294 (32.0%)	2.38	1.39	
Retired	38 (4.1%)	2.71	1.41	
Monthly income				0.370
<5000 SR	127 (13.8%)	2.35	1.53	
5000–15,000 SR	451 (49.0%)	2.43	1.28	
>15,000 SR	343 (37.2%)	2.53	1.30	

*p*-Value: One Way ANOVA; ^$^: Independent t-test; * *p* < 0.05 (significant).

**Table 2:** Distribution of participants’ level of knowledge about the potential harms of foods sold by the productive families-owned food trucks.

Knowledge Items	Correct		Incorrect		Don’t Know	
	Number	%	Number	%	Number	%
Abortion in pregnant women can be induced by food-borne disease.	464	50.4%	201	21.8%	256	27.8%
Hepatitis A virus is a food-borne pathogen.	570	61.9%	126	13.7%	225	24.4%
AIDS can be transmitted by food.	169	18.3%	592	64.3%	160	17.4%
Buying from family producer-owned food trucks increases the risk of food poisoning.	498	54.1%	277	30.1%	146	15.9%
Reheating cooked foods can contribute to food contamination.	562	61.0%	183	19.9%	176	19.1%
Overall knowledge (Mean ± SD)				2.5 ± 1.2 (52%)		

**Table 3:** Multiple linear regression model for determinants of consumers’ knowledge regarding potential harms of foods sold by the food trucks.

Factors	Unstandardized Coefficients		Standardized Coefficients	t	*p*-Value
	B	SE	Beta		
Age in years Female	−0.05 −0.10	0.06	−0.04	−0.84	0.400
		0.09	−0.04	−1.08	0.281
Married	1.40	0.10	1.10	4.10	0.001 *
Saudi	0.41	0.22	0.06	1.87	0.061
High educational level	−0.12	0.08	−0.05	−1.62	0.043 *
Healthcare workers	1.30	0.06	1.10	0.17	0.868
Monthly income	0.50	0.07	0.31	1.79	0.074

B: Regression coefficient; SE: Standard error; * *p* < 0.05 (significant).

**Table 4:** Distribution of consumers’ attitude towards the safety of foods sold by the food trucks.

	Attitude towards Safety of Foods Sold by Food Trucks						*p*-Value
	Negative		Neutral		Positive		
	Number	%	Number	%	Number	%	
Age in years							0.507 ^$^
18–25	17	3.5%	152	31.5%	313	64.9%	
26–34	4	1.8%	81	37.3%	132	60.8%	
35–44	4	3.0%	39	29.1%	91	67.9%	
45–54	2	2.9%	22	31.4%	46	65.7%	
55+	0	0.0%	3	16.7%	15	83.3%	
Gender							0.104
Male	15	3.9%	113	29.1%	260	67.0%	
Female	12	2.3%	184	34.5%	337	63.2%	
Marital status							0.346
Single	20	3.4%	192	32.7%	376	63.9%	
Married	4	1.4%	90	32.0%	187	66.5%	
Divorced/widow	3	5.8%	15	28.8%	34	65.4%	
Nationality							0.963
Saudi	26	2.9%	284	32.2%	573	64.9%	
Non-Saudi	1	2.6%	13	34.2%	24	63.2%	
Educational level							0.523
Below secondary	0	0.0%	11	25.0%	33	75.0%	
Secondary	8	3.2%	84	34.0%	155	62.8%	
University/above	19	3.0%	202	32.1%	409	64.9%	
Job title							0.219
Not employed	4	3.2%	44	34.9%	78	61.9%	
Non-health care workers	12	2.6%	148	32.0%	303	65.4%	
Healthcare workers	11	3.7%	99	33.7%	184	62.6%	
Retired	0	0.0%	6	15.8%	32	84.2%	
Monthly income							0.021 *
<5000 SR	6	4.7%	36	28.3%	85	66.9%	
5000–15,000 SR	16	3.5%	163	36.1%	272	60.3%	
>15,000 SR	5	1.5%	98	28.6%	240	70.0%	

^$^: Exact probability test; * *p* < 0.05 (significant).

**Table 5:** Multiple logistic regression model for determinants of participants’ attitude towards the safety of foods sold by the food trucks.

Factors	*p*-Value	OR	95% CI	
			Lower	Upper
Age Female	0.571 0.433	1.05	0.88	1.26
		0.90	0.70	1.17
Married	0.678	1.07	0.79	1.45
Saudi	0.676	1.12	0.65	1.93
High education	0.647	0.95	0.77	1.17
Healthcare workers	0.301	1.10	0.92	1.31
Income	0.047 *	1.19	1.02	1.45

OR: Odds ratio; CI: Confidence interval; * *p* < 0.05 (significant).

## Data Availability

The data presented in this study are available within the article.

## Supplementary Materials

The following supporting information can be downloaded at: https://www.mdpi.com/article/10.3390/ijerph19074322/s1, File S1. Survey questions on Food Safety Knowledge and Attitudes among Saudi Consumers from Food Trucks Owned by Family Producers.
