# Supplementary material for: Food Safety Knowledge and Attitudes: A Cross-Sectional Study among Saudi Consumers from Food Trucks Owned by Productive Families
Source: Int J Environ Res Public Health. 2022 Apr 4;19(7):4322. doi: 10.3390/ijerph19074322 (PMC8998948; doi:10.3390/ijerph19074322)
Supplement: Supplementary file 1 [file ijerph-19-04322-s001.zip › ijerph-1635602-supplementary.pdf]

Survey Questionnaire

Title: Food safety knowledge and attitudes: A cross-sectional study among Saudi consumers of food trucks owned by productive families

Part 1: Study objectives, consent letter and screening question [At the beginning of the survey link]

Hi, we are a research team at King Faisal University and are conducting a research study to assess the knowledge and attitude of consumers of productive families-owned FTs regarding food safety and foodborne disease.

This survey will take approximately 15 minutes to complete. If you choose to participate, you will be asked to complete a survey with questions about your demographic characteristics such as gender, age employment status, income. etc., and then some questions to assess your food safety knowledge and attitude about the food provided by FTs will be asked. Your responses will be anonymous. No one will be able to identify the respondent. Sensitive information such as name, mailing address, social security number, or phone number are not asked. Only the researchers involved in this study and those responsible for research oversight will have access to the information you provide.

Participation in this study is completely voluntary. You can decline to participate or end participation at any time for any reason. Your responses will be stored in a secure system of this survey website. Once the research is complete, the stored data will be destroyed from this website. The survey data used for our analysis will be stored in a locked hardware with a password and a locked office for 5 years after the termination of the study.

If you have any questions about this study, you may contact the principal investigator (Dr. Najim Alshahrani, email: nalshahrani@uj.edu.sa).

By clicking "I agree" below, you are indicating that you are at least 18 years old, have read and understood this consent form, and agree to participate in this research study. Please print a copy of this page for your records.

- I agree
- I do not agree

Did you ever buy ready-to-eat food from Food Trucks made by Saudi productive families?

- Yes. [Then continue to the survey]
- No. [Send response without being included in the survey]

Part 2: Demographic Characteristics of the study participants

1. What is your gender?	(a)Male (b) Female
2 What is your age (years)?	………………
3. Occupation	(a) Student (b) Business (c) Unemployment<br>(d) Employer in health sector (e.g: doctor, nurse)<br>(e) Employer in non-helath sector (e.g: teacher) (g) Retired<br>(H) others
4. Education level	(a) No Formal Education (b) Primary (C) Secondary (d) University or above
5. Marital status	(a) Single (b) Married
6 Household income	(a) Less than 5,000 R<br>(b) 5,000 – 15, 000 R<br>(c) More than 15,000 R

Part 3: Assessment of the food safety knowledge of consumers of food trucks owned by productive families

Statement	Correct	Incorrect	Don't know
1. Abortion in pregnant women can be induced by food-borne disease.			
2. Hepatitis A virus is a food-borne pathogen.			
3. Buying from food trucks of productive families increases the risk of food poisoning.			
4. AIDS can be transmitted by food.			
5. Reheating cooked foods can contribute to food contamination.			

Part 4: Assessment of food safety attitudes of consumers

Questions	Agree	Disagree	No idea
1. Proper hand hygiene can prevent food-borne diseases.			
2. Raw and cooked foods should be stored separately to reduce the risk of food contamination.			
3. The health status of workers should be evaluated before employment.			
4. The best way to thaw a chicken is in a bowl of cold water.			
5. Wearing masks is an important practice to reduce the risk of food contamination.			
6. Wearing gloves is an important practice to reduce the risk of food contamination.			
7. Wearing caps is an important practice to reduce the risk of food contamination.			
8. Dish towels can be a source of food contamination.			
9. Knives and cutting boards should be properly sanitized to prevent cross-contamination.			
10. Food handlers who have abrasions or cuts on their hands should not touch foods without gloves.			
11. Well-cooked foods are free of contamination.			
12. Can a closed can/jar of cleaning product be stored together with closed cans and jars of food products.			
13. The ideal place to store raw meat in the refrigerator is on the bottom shelf.			
